# Sex-specific colonic mitochondrial dysfunction in the indomethacin-induced rat model of inflammatory bowel disease

**DOI:** 10.3389/fphys.2024.1341742

**Published:** 2024-03-26

**Authors:** Ngoc Hoang, Karen Brooks, Kristin Edwards

**Affiliations:** Department of Pharmacology and Toxicology, University of Mississippi Medical Center, Jackson, MS, United States

**Keywords:** inflammatory bowel disease, mitochondria, oxidative phosphorylation, reactive oxygen species, mitochondrial-targeted therapies, MitoTEMPO

## Abstract

**Introduction:** Inflammatory bowel disease (IBD) is characterized by chronic inflammation of the gastrointestinal tract and encompasses Crohn’s Disease and Ulcerative Colitis. Women appear to have more severe and recurring symptoms of IBD compared to men, most likely due to hormonal fluctuations. Studies have shown that mitochondrial dysfunction plays a role in the development of inflammation and there is evidence of colon mitochondrial alterations in IBD models and patients. In this study we have identified the presence of sex-specific colon mitochondrial dysfunction in a rat model of IBD.

**Methods:** Eight-week-old male and female rats were treated with indomethacin to induce IBD and mitoTEMPO was administered daily either after or before induction of IBD and until euthanasia. Colons were collected for histology and mitochondrial experiments. Intact mitochondrial respiration, reactive oxygen species (mtROS), the activities of the individual electron transport complexes and the activities of the antioxidant enzymes were measured to assess mitochondrial function.

**Results:** IBD male rats showed a decrease in citrate synthase activity, cardiolipin levels, catalase activity and an increase in mtROS production. IBD females show a decrease in intact colon mitochondrial respiration, colon mitochondria respiratory control ratio (RCR), complex I activity, complex IV activity, and an increase in mtROS. Interestingly, control females showed a significantly higher rate of complex I and II-driven intact mitochondrial respiration, MCFA oxidation, complex II activity, complex III activity, and complex IV activity compared to control males. The use of a mitochondrial-targeted therapy, mitoTEMPO, improved the disease and colon mitochondrial function in female IBD rats. However, in the males there was no observed improvement, likely due to the decrease in catalase activity.

**Conclusion:** Our study provides a better understanding of the role mitochondria in the development of IBD and highlights sex differences in colon mitochondrial function. It also opens an avenue for the development of strategies to re-establish normal mitochondrial function that could provide more options for preventive and therapeutic interventions for IBD.

## 1 Introduction

In the US, approximately three million people suffer from inflammatory bowel disease (IBD), with the highest prevalence in the non-Hispanic white population (Xu et al., 2018). IBD is an umbrella term for two chronic inflammatory conditions, Crohn’s Disease (CD) and Ulcerative Colitis (UC) (MKoma, 2013). Both of these conditions are characterized by chronic inflammation of the gastrointestinal (GI) track (MKoma, 2013). CD can affect any part of the GI tract from the mouth to the anus, but most often, it affects the portion of the small intestines before the large intestine/colon. The damaged areas usually appear as inflamed patches that are next to areas of healthy tissue. Inflammation may reach through the multiple layers of the GI tract that can result in blockages of the GI track requiring surgical removal (Xu et al., 2018). UC occurs only in the large intestines and rectum. Inflammation is continuous usually starting in the rectum and spreading further into the colon. Inflammation is present only in the innermost layer of the lining of the colon. Chronic inflammation of the colon leads to a higher risk for colorectal cancer (CRC). Currently, there is no known cure for IBD with treatments mainly focusing on reducing inflammation and symptoms (MKoma, 2013).

To study IBD, many rodent models are available and all have advantages and disadvantages (Kiesler et al., 2001). In particular, indomethacin-induced IBD in rats is a well-established model. Indomethacin is an easily administered, widely available, and clinically relevant compound that induces distinct acute and chronic phases of IBD that involve both the small and large intestine (Kiesler et al., 2001). This model also has an association with extraintestinal lesions as well as differences in genetic susceptibility among inbred rat strains. This model has been very useful in identifying the role of various factors involved in the pathogenesis of intestinal inflammation and should provide a reliable and noninvasive model for drug screening (Kiesler et al., 2001).

GI inflammation is a result of decreased intestinal functionality associated with epithelial barrier dysfunction (Ballard and Towarnicki, 2020; Jackson and Theiss, 2020). The epithelial barrier refers to the property of the intestinal mucosa that ensures adequate containment of undesirable luminal contents within the intestine while preserving the ability to absorb nutrients. Its role is to protect the gut mucosal tissues and circulatory system from exposure to pro-inflammatory molecules, such as microorganisms, toxins, and antigens that are vital for the maintenance of health and wellbeing (Ballard and Towarnicki, 2020). Mitochondria play an essential role in the maintenance of the epithelial barrier (Ballard and Towarnicki, 2020; Jackson and Theiss, 2020). Mitochondria are the organelles responsible for energy production within the cell. Mitochondrial dysfunction, which results in an increase in mitochondrial reactive oxygen species (mtROS), is associated with intestinal disease and the development of colorectal cancer (Restivo et al., 2004; Sifroni et al., 2010; Santhanam et al., 2012a; Santhanam et al., 2012b; Ussakli et al., 2013; Kolli et al., 2014; Novak and Mollen, 2015; Berger et al., 2016; Khaloian et al., 2020; Smith et al., 2021). A proposed mechanism is that mtROS increases in the GI epithelial cells, this will target the commensal bacteria leading to gut dysbiosis, decrease substrate availability for the epithelial cells, decreased ATP production, and a decrease in the mucosal layer. These changes induce leakage of pathogen-associated molecular patterns (PAMPs) that trigger inflammation (Ballard and Towarnicki, 2020). Many studies show evidence of mitochondrial dysfunction in IBD models and human samples (Restivo et al., 2004; Sifroni et al., 2010; Santhanam et al., 2012a; Santhanam et al., 2012b; Ussakli et al., 2013; Kolli et al., 2014; Wang et al., 2014; Novak and Mollen, 2015; Berger et al., 2016; Heller et al., 2017; Jackson et al., 2020; Khaloian et al., 2020; Smith et al., 2021). There has also been evidence mitochondrial complex IV is altered in UC and can lead to the development of CRC (Ussakli et al., 2013). However, further investigation into the mechanism(s) by which mitochondria function induces the development of intestinal diseases is needed.

Sex differences are observed in IBD. Both CD and UC show sex differences in disease characteristics (Goodman et al., 2020). For example, both the incidence and severity of CD are reportedly increased in female patients compared to males, and female gender is a risk factor for relapse in UC (Xu et al., 2018; MKoma, 2013; Goodman et al., 2020). Female patients experience dichotomous changes in disease symptoms during puberty, pregnancy and menopause (Goodman et al., 2020). Genome linkage studies have revealed high frequency haplotypes in chromosome X associated with IBD in females, although the specific genetic loci that confer this bias remain elusive (Masayuki Saruta et al., 2009). Interestingly, mitochondria are also regulated by sex hormones via the estrogen and androgen receptors, which can lead to differences in mitochondrial function between males and females (Liao et al., 2015; Bajpai et al., 2019). However, most studies investigating IBD rat models use only one sex. Therefore, mechanistic investigations into the role of sex in the development of IBD are limited. Understanding the link between sex, mitochondrial function, and the development of IBD will provide additional information for the development of therapeutic options.

Currently there are various mitochondrial-targeted therapies that could prove useful. One in particular, mitoTEMPO, a superoxide dismutase (SOD) mimetic, has been shown to improve epithelial barrier function, to reduce bacteria internalization and transcytosis, to reduce colitis in the dextran sulfate sodium (DSS) mouse model of UC, and to reduce DSS-induced proinflammatory cytokine (Wang et al., 2014). MitoTEMPOL, another SOD mimetic but with a slightly different structure, combined with a biocompatible B-cyclodextrin-derived material has been shown to improve the disease in both acute and chronic mouse models of IBD (Zhang et al., 2016). Therefore, mitochondrial-targeted therapy proves to be a potential treatment option for IBD patients.

The goals of the present study were (1) to investigate if colon mitochondrial dysfunction and increased mitochondrial ROS (mtROS) is present in the indomethacin-induced model of IBD; (2) to determine if any sex differences are observed in the mitochondrial dysfunction; and (3) to determine if treatment with the mitochondrial-targeted therapy, mitoTEMPO, would improve the disease activity.

## 2 Materials and methods

### 2.1 Animals

Animals were purchased from Harlan Laboratory at 7 weeks of age and allowed to acclimate for at least 1 week before use. All animals were housed in the Center for Comparative Research animal facilities of the University of Mississippi Medical Center (UMMC). Animals were kept on a 12 h light/12 h dark cycle and fed a standard laboratory rodent diet (Teklad 8640). All procedures were conducted in accordance with the National Institutes of Health’s Guide for the Care and Use of Laboratory Animals and approved by the Institutional Animal Care and Use Committee.

### 2.2 Indomethacin-induced inflammatory bowel disease

Male and female Sprague Dawley rats at 8 weeks of age were randomly assigned to either control or the indomethacin group. Indomethacin (7.5 mg/kg) was administered to the indomethacin group by subcutaneous injection once a day for 2 days, exactly 24 h apart (Kiesler et al., 2001). Control rats received saline/ethanol. Indomethacin is known to cause small intestinal and colonic ulcerations (Kiesler et al., 2001). The peak for the disease is reported to be 2–3 days after indomethacin treatment with a duration up to 7 days. However, it has also be reported that the elevation of inflammatory markers can be observed for up to 90 days (Porras et al., 2004). Therefore, animals were euthanized on day 2, day 3, day 7, and day 30 for experiments to determine the peak of the disease. Animal body weight along with food intake was measured daily. For mitoTEMPO treatment groups, rats were randomly assigned into control, control + mitoTEMPO, indomethacin, and indomethacin + mitoTEMPO groups. MitoTEMPO (1 mg/kg) was injected intraperitoneal daily either at the start of indomethacin treatment (prophylaxis protocol) or after the indomethacin injections (treatment protocol). Total disease activity score was determined using the sum of the score for weight loss (0 = ≤ 2%; 1 = 3–6%; 2 = 7–12%; 3 = > 12%), stool consistency (0 = Normal Stool; 1 = Semi-solid Stool; 2 = Loose to Pasty Stool; 3 = Diarrhea), and color of stool (0 = brown; 1 = dark brown; 2 = black; 3 = bloody). Colon lengths and weights were also measured upon removal as another indicator of disease.

### 2.3 Histology

Colon swiss rolls were prepared similar to previously described methods (Bialkowska et al., 2016). In brief colons were washed with modified Bouin’s fixative (50% ethanols/5% acetic acid in dH_2_O) using a 10 mL syring with a gavage needle. The colon was cut open and placed on a Petri dish with the luminal side facing upward and rinsed with PBS. The colon is then gently rolled around a toothpick and placed in 10% formalin overnight. The tissues were embedded in paraffin, and serially cut into 4-µm-thick sections and stained with hematoxylin and eosin at the Histology Core located within the Department of Physiology and Biophysics at the University of Mississippi Medical Center. Microscopic images were taken at × 2 using an Olympus BX63.

### 2.4 Colon tissue homogenate

The remaining colon tissue was weighed and placed in isolation buffer (200 mM mannitol, 50 mM sucrose, 5 mM MOPS, pH 7.15, 0.1% BSA) for further cleaning to ensure removal of feces. The colon was then incubated with 1 mL of Bacterial Proteinase XXIV (Sigma, 2 mg/mL) for 5 min. The colon was removed from proteinase and incubated with homogenization buffer (200 mM mannitol, 50 mM sucrose, 5 mM MOPS, pH 7.15, 20 mg/mL BSA, 1 mM EDTA, 0.2 mM PMSF). The colon was then cut into small pieces and homogenized gently for five strokes in a Potter-Elvehjem homogenizer where the pestle was shaved to reduce the diameter to 1 mm. The tissue homogenate was used immediately for intact mitochondrial experiments and mtROS experiments. After finishing the assay, colon tissue homogenates were fast-frozen in liquid nitrogen and used for further mitochondrial enzyme experiments.

### 2.5 Intact mitochondria assays

Intact mitochondria respiration was measured using the Oroboros O2k FluoRespirometer at 37°C by previously published methods (Shirey et al., 2016; Edwards et al., 2018). Briefly, each chamber of the O2k was calibrated for the O_2_ concentration in nano-pured water. For complex I-driven and complex II-driven respiration, reaction mixture included buffer containing 10 mM KPi, 5 mM MgCl_2_, 30 mM KCl, 1 mM EDTA, and 75 mM Tris, pH 7.5 with additions of 7.3 µM cytochrome *c* and either 20 mM glutamate and 5 mM malate or 20 mM succinate. The total volume of the assay mixture was 2.1 mL. State 2 respiration were initiated by injecting 100 µL fresh colon tissue homogenate. State three respiration (ATP synthesis) is achieved by adding 2 mM ADP. The respiratory control ratio (RCR) was calculated by the ratio of state three to state 2 respiration. For fatty acid oxidation, the reaction was performed in the same assay buffer as above with 0.5 mM malate, 2 mM ATP, 1 mM NAD^+^, 25 µM cytochrome *c*, 0.1 mM coenzyme A, and either 0.5 mM oleate (long-chain fatty acid, LCFA) or 0.5 mM octanoate (medium-chain fatty acid, MCFA). The reaction was started by injecting 100 µL of fresh colon tissue homogenate. For LCFA oxidation, 2 mM carnitine was added to support the transport of LCFA through the inner mitochondrial membrane.

### 2.6 Mitochondrial ROS measurement

Mitochondrial ROS (mtROS) was measured using the Amplex Red assay, which measures the production of H_2_O_2_^28^. Amplex Red (10 µM) is converted to resorufin (highly fluorescent) by the reaction of H_2_O_2_ and horseradish peroxidase (HRP, 1U/mL). Superoxide dismutase (SOD, 5 U/mL) was also added to convert superoxide (SO) to H_2_O_2_. The rate of H_2_O_2_ production was obtained by calibrating the fluorescence response of known concentrations of H_2_O_2_ in the Oroboros O2k FluoRespirometer. By stoichiometry, the rate of SO production is twice the rate of H_2_O_2_ production. Therefore, we calculated the parameter of ‘percent electron leak’, defined as the percentage of electrons from NADH or succinate that are shunted to SO, as opposed to flowing to O_2_ in Complex IV. Expression of ROS production as ‘percent electron leak’ enhances the comparisons of mtROS production induced in different animals.

### 2.7 Mitochondrial content

Citrate synthase activity and cardiolipin are reported as the best markers for mitochondrial content (Larsen et al., 2012). Citrate synthase activity was measured via production of coenzyme A (CoASH) from oxaloacetate. In the presence of 5,5′-dithio-bis- (2-nitobenzoic acid) (DTNB) and free sulfhydryl groups of coenzyme A react to form free 5-thio-2-nitrobenzoate anions which were measured at 412 nm (ε = 13.6 mM^–1^cm^–1^) using plate reader SpectraMax M5. The reaction mixture included 1 mM DNTB, 0.3 mM acetyl CoA, 1% sodium cholate, 200 µL of colon tissue homogenates. The reaction was initiated by adding 0.5 mM oxaloacetate. The rates were normalized to gram of tissue used to prepare the homogenate. The citrate synthase rates were used to normalize all mitochondrial rates to mitochondrial content. Cardiolipin was measured using the Abcam Cardiolipin Assay Kit (ab241036). Colon tissue homogenates were prepared and the assay was performed as outlined in the kit manual.

### 2.8 Individual mitochondrial enzymes

Complexes I, II, and III activities were measured using a Jasco UV-Vis spectrophotometer at 25°C following previously published methods (Shirey et al., 2016; Edwards et al., 2018). Briefly, *complex I* activity was measured as the time-dependent oxidation of NADH at 340 nm using a measured extinction coefficient of 6.22 mM^–1^cm^–1^. The reaction mixture in a regular quartz cuvette contained 10 mM KPi, 5 mM MgCl_2_, 30 mM KCl, 1 mM EDTA, 75 mM Tris, pH 7.5, 100 µM Coenzyme Q1 (CoQ1), 5 µM antimycin A, and 100 µL colon tissue homogenates in a final volume of 2 mL. The reaction was initiated by adding 100 µM NADH and the fastest rates were obtained after NADH was added. *Complex II* activity was measured as the time-dependent oxidation of 2,6-dichloroindophenol sodium salt hydrate (DCPIP) at 600 nm using a measured extinction coefficient of 19.1 mM^–1^cm^–1^. The reaction mixture in a micro-cuvette contained 10 mM KPi, 5 mM MgCl_2_, 30 mM KCl, 1 mM EDTA, 75 mM Tris, pH 7.5, 20 mM succinate, 80 µM DCPIP, 1 mM KCN, 5 µM Antimycin A, 4 µM rotenone, and 20 µL of colon tissue homogenates in a final volume of 200 µL. Reactions were initiated by adding 50 µM decylubiquinone and the linear rates were obtained. *Complex III* activity was measured as the time-dependent reduction of cytochrome *c* at 550 nm using a measured extinction coefficient of 18.7 mM^–1^cm^–1^. The reaction mixture in a micro-cuvette contained 10 mM KPi, 5 mM MgCl_2_, 30 mM KCl, 1 mM EDTA, 75 mM Tris, pH 7.5, 3 mM KCN, 4 µM rotenone, 60 µM cytochrome *c* (LeeBio, cat # 192-10-1), 20 µL of colon tissue homogenates in a final volume of 200 µL. Reactions were initiated with 100 µM reduced decylubiquinone (Fisher Scientific, cat # NC1291121). The rate was linear for 1–2 min. *Complex IV* activity was measured at 37°C as the rate of oxygen consumption using an Oroboros FluoRespirometer. Ascorbate and N,N,N′,N′-Tetramethyl-p-phenylenediamine dihydrochloride (TMPD) were used to donate electrons to soluble cytochrome *c*. Reaction mixture contained 50 mM Tris, 8 mM KCl, 1 mM EDTA, 16 μg/μL catalase, pH 7.4, 3 mM ascorbate, 0.3 mM TMPD, 25 µM cytochrome *c.* Reactions were initiated by addition of 100 µL of thawed colon tissue homogenates in a final volume of 2 mL. For each enzyme, the inhibited non-enzymatic background rates were subtracted from the enzymatic rates.

### 2.9 Antioxidant enzyme measurements

The activity of the antioxidant enzymes glutathione peroxidase (GPx), glutathione S-transferase (GST), Glutathione reductase (GR), catalase (CAT), superoxide dismutase (SOD) and glutathione (GLT) levels were determined using colorimetric assay kits from Cayman Chemical following the manufacturer’s protocols.

### 2.10 Inflammatory marker measurements

The presence of c-reactive protein (CRP) and inflammatory cytokines interleukin -1β (IL-1β) and tumor necrosis factor alpha (TNFα) were measured in the serum of rats using ELISA kits from Abcam following the manufacturer’s protocols.

### 2.11 Statistics

Data are expressed as mean ± SE. Comparisons between two groups were performed using unpaired Student’s t-test. Comparisons between multiple experimental groups were made by a one-way ANOVA analysis. Statistical significance is indicated as a *p*-value ≤0.05. All studies had an n ≥ 4. All analyses were performed with GraphPad Prism version 9.0.

## 3 Results

### 3.1 Indomethacin-induced IBD model

**FIGURE 1:**
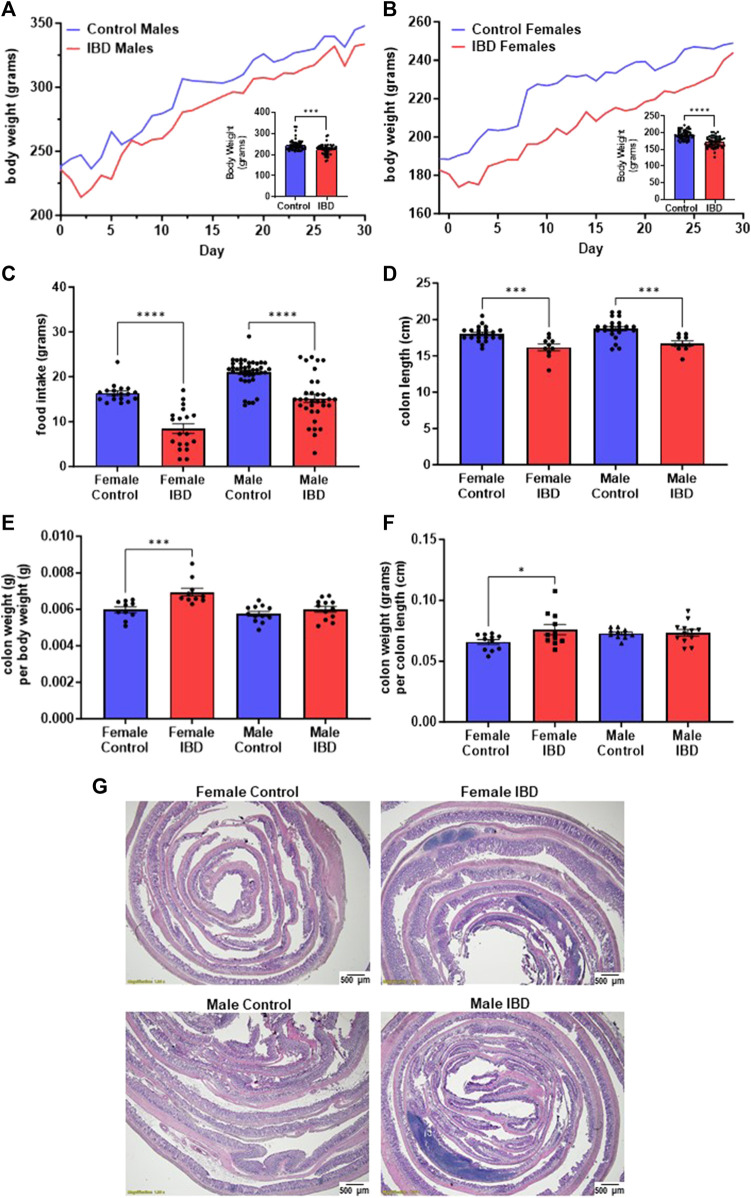
Indomethacin-induced IBD decreases body weight, food intake, and colon length while showing an increase in colon inflammation at day 2–3 following injections. The body weight of male **(A)** and female **(B)** rats was monitored daily for 30 days. The peak of the disease is at day 2–3. The inserts show a significant decrease in body weight at the peak of the disease for both males (*p* ≤ 0.001) and females (*p* ≤ 0.0001). **(C)** The food intake of these animals was also significantly decreased at the peak of the disease (days 2–3; *p* ≤ 0.0001). **(D)** Colon length was significantly decreased in both male and female IBD rats at days 2–3 (*p* ≤ 0.001). **(E)** Colon weight divided by the body weight was used as an indicator for potential hypertrophy which was significantly increased in females at days 2–3 (*p* ≤ 0.001). **(F)** Another indicator of potential hypertrophy is colon weight divided by colon length which was again significantly increased in females at days 2–3 (*p* ≤ 0.001). **(G)** Histology with H&E staining showed increased areas of inflammation in males and females at day 2.

One of the key indicators of the induction of IBD by indomethacin is the decline of body weight at the peak of the disease, which is reported to be 2–3 days following treatment (Kiesler et al., 2001). In our animals, we observed a significant decline of body weight on day 2 and day 3 (Figures 1A, B) following injection with their body weight recovering over time. Males showed an 8% loss in body weight at the peak of the disease (Figure 1A). Females showed a more significant loss of body weight (11%, Figure 1B). This loss in body weight could be attributed to the significant decrease in food intake observed at the peak of the disease (Figure 1C). Another indicator of indomethacin induced IBD is the decrease in colon lengths. In Figure 1D, both male and female IBD rats showed a significant decrease in colon length at the peak of the disease. This decrease was returned to normal at day 7 and day 30 (Supplementary Figures S1A, B). Only the females showed a significant increase in colon weight to body weight ratio at the peak of the disease, that returns to normal at day 7 and 30 (Figure 1E, Supplementary Figures S1C, D). Additionally, only females showed a significant increase in colon weight to colon length ratios at the peak of the disease that again recovered at day 7 and day 30 (Figure 1F, Supplementary Figures S1E, F). Another indicator of indomethacin induced IBD is the increase in GI inflammation. In our animals, we observed a significant increase in areas of infiltration in the colons at the peak of the disease with females being higher (Figure 1G). Taken together these results indicate that indomethacin induced an acute GI inflammatory response that peaked at day 2–3 following treatment with only females showing indications of hypertrophy at the peak of the disease. Therefore, our mitochondrial studies primarily focus on days 2–3 following indomethacin injections.

### 3.2 Mitochondrial content decreases in IBD male rats

**FIGURE 2:**
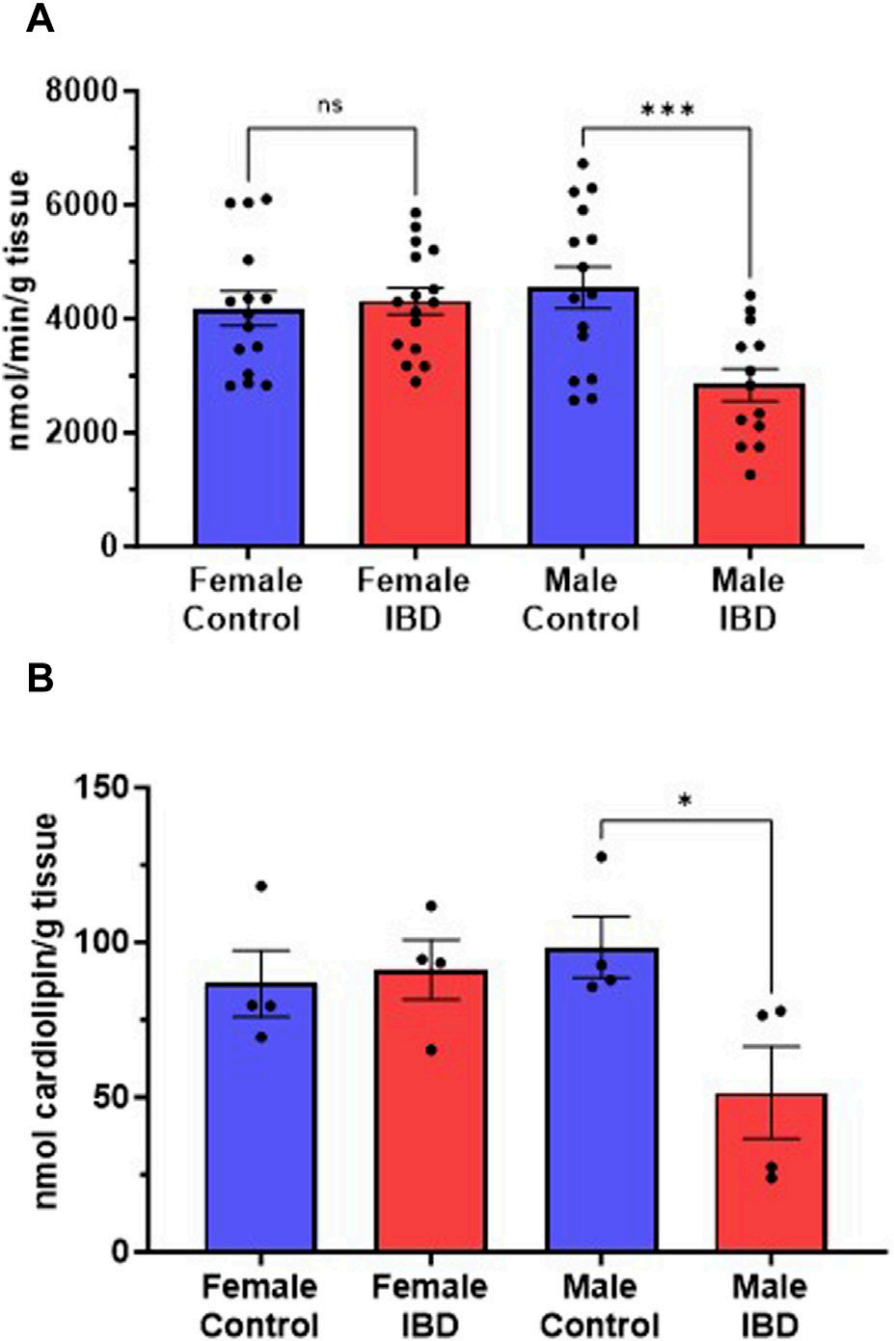
**(A)** Citrate synthase activity is a reliable marker of mitochondrial content. In our colon tissue homogenate samples, we saw a significant decrease in citrate synthase activity in males (*p* ≤ 0.001), but not females at days 2–3. **(B)** Cardiolipin levels, another marker for mitochondrial content, showed a similar result at day 2 (*p* ≤ 0.05). Mitochondrial respiration was normalized to mitochondrial content using citrate synthase activity.

Citrate synthase activity and cardiolipin levels were used as markers for mitochondrial content in the tissue homogenate samples (Larsen et al., 2012). Indomethacin-induced IBD resulted in a significant loss of citrate synthase activity in male colon samples but not females (Figure 2A). A similar trend was observed when measuring cardiolipin levels (Figure 2B). Therefore, we relied on citrate synthase activity to normalize mitochondrial activities to mitochondrial content. This normalization allows us to conclude that any observed differences in activity are not due to changes in the number of mitochondria in the colon.

### 3.3 Intact mitochondrial respiration decreases in IBD female rats

**FIGURE 3:**
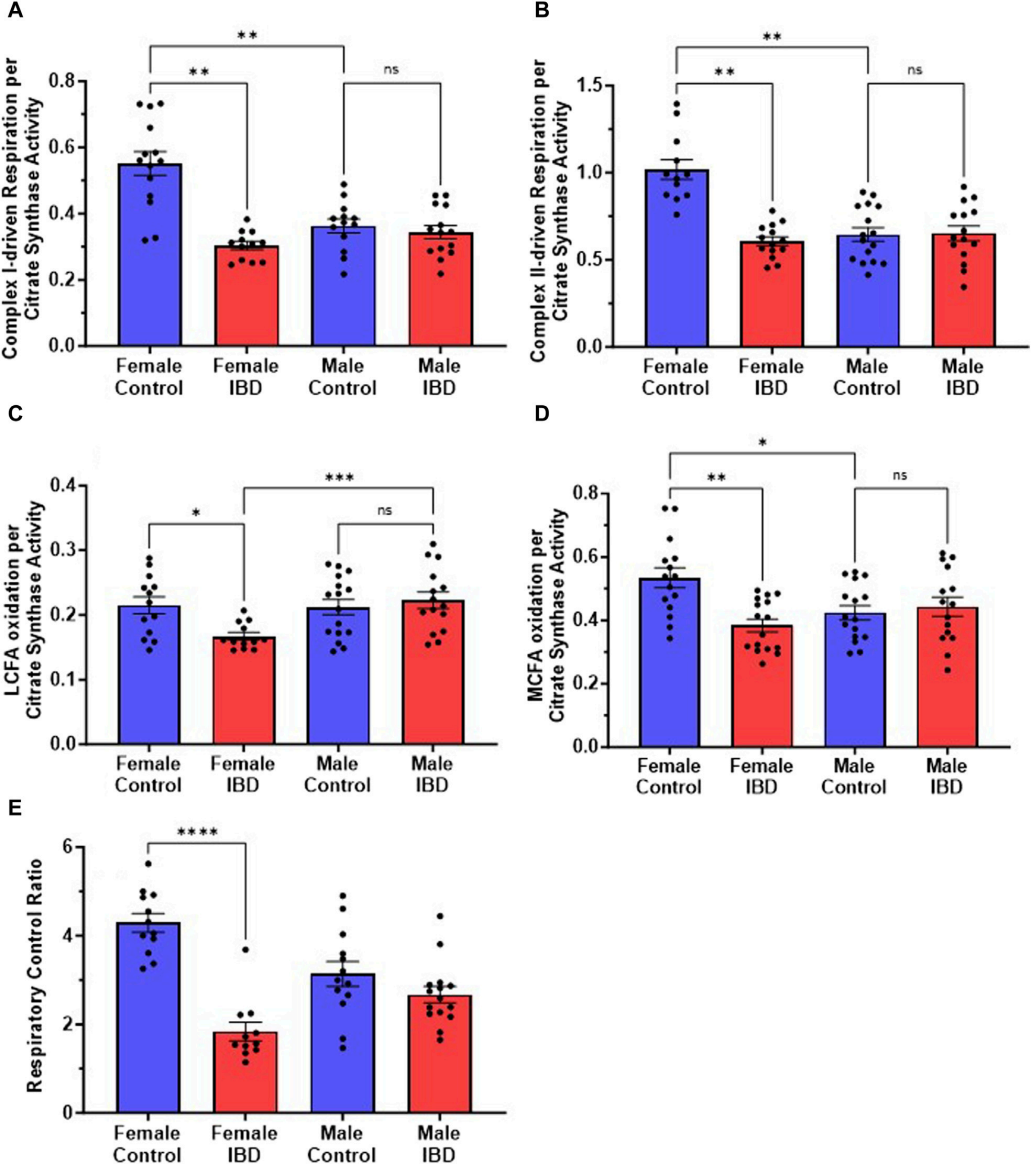
**(A)** Complex I-driven mitochondrial respiration (20 mM glutamate and 10 mM malate plus 2 mM ADP) shows a significant decrease in females with IBD (days 2–3) compared to controls (*p* ≤ 0.01). No significant difference was observed in males with IBD compared to controls. Male controls were significantly lower compared to females (*p* ≤ 0.05). **(B)** Complex II-driven mitochondrial respiration (20 mM succinate plus 2 mM ADP) shows a significant decrease in activity in females with IBD (days 2–3) compared to controls (*p* ≤ 0.01). No significant difference was observed in males with IBD compared to controls. Male controls were significantly lower compared to females (*p* ≤ 0.05). **(C)** LCFA oxidation (0.5 mM Oleate plus 2 mM carnitine) shows a significant decrease in activity in females with IBD (days 2–3) compared to controls (*p* ≤ 0.05). No significant difference was observed in males. **(D)** MCFA oxidation (0.5 mM octanoate) shows a significant decrease in activity in females with IBD (days 2–3) compared to controls (*p* ≤ 0.01). No significant difference was observed in males. Male controls were significantly lower compared to females (*p* ≤ 0.05). **(E)** The respiratory control ratio (RCR) is an indicator of the quality of mitochondria. This was determined as outlined in methods. A significant decrease in RCR was seen only in females (*p* ≤ 0.001) at days 2–3, which suggests poorly coupled mitochondria. Respiration rates were normalized to citrate synthase activity with final units of nmol e-/nmol citrate.

Our initial exploration showed the mitochondrial function was significantly decreased in females at the peak of the disease, but full activity returned at day 7 and 30 (Supplementary Figure S2A) with no change on any day with the males (Supplementary Figure S2B). Therefore, for the remaining experiments rats were used on day 2 or 3 following treatment. When examining the rates of respiration, the colon mitochondria from female rats with IBD showed a significant decrease in complex I-driven respiration (Figure 3A), complex II-driven respiration (Figure 3B), LCFA oxidation (Figure 3C), and MCFA oxidation (Figure 3D) when normalized to mitochondrial content. However, the males showed no significant change when normalized to mitochondrial content. Interestingly, control females had significantly higher complex I and complex II-driven colonic mitochondrial respiration as well as MCFA oxidation compared to control males (Figures 3B, C, E).

The quality of intact mitochondria is determined by measuring the respiratory control ratio (RCR) as described in the methods. The RCR for these experiments was derived from the respirations rates with and without the addition of ADP. The higher the ratio the more intact the mitochondria resulting in better production of ATP. For colon mitochondria, controls had an average RCR of 4 (Figure 3E). IBD females showed a significant decrease in RCR to an average of 2 with no significant difference seen in IBD males (Figure 3E).

### 3.4 mtROS production increases in IBD rats

**FIGURE 4:**
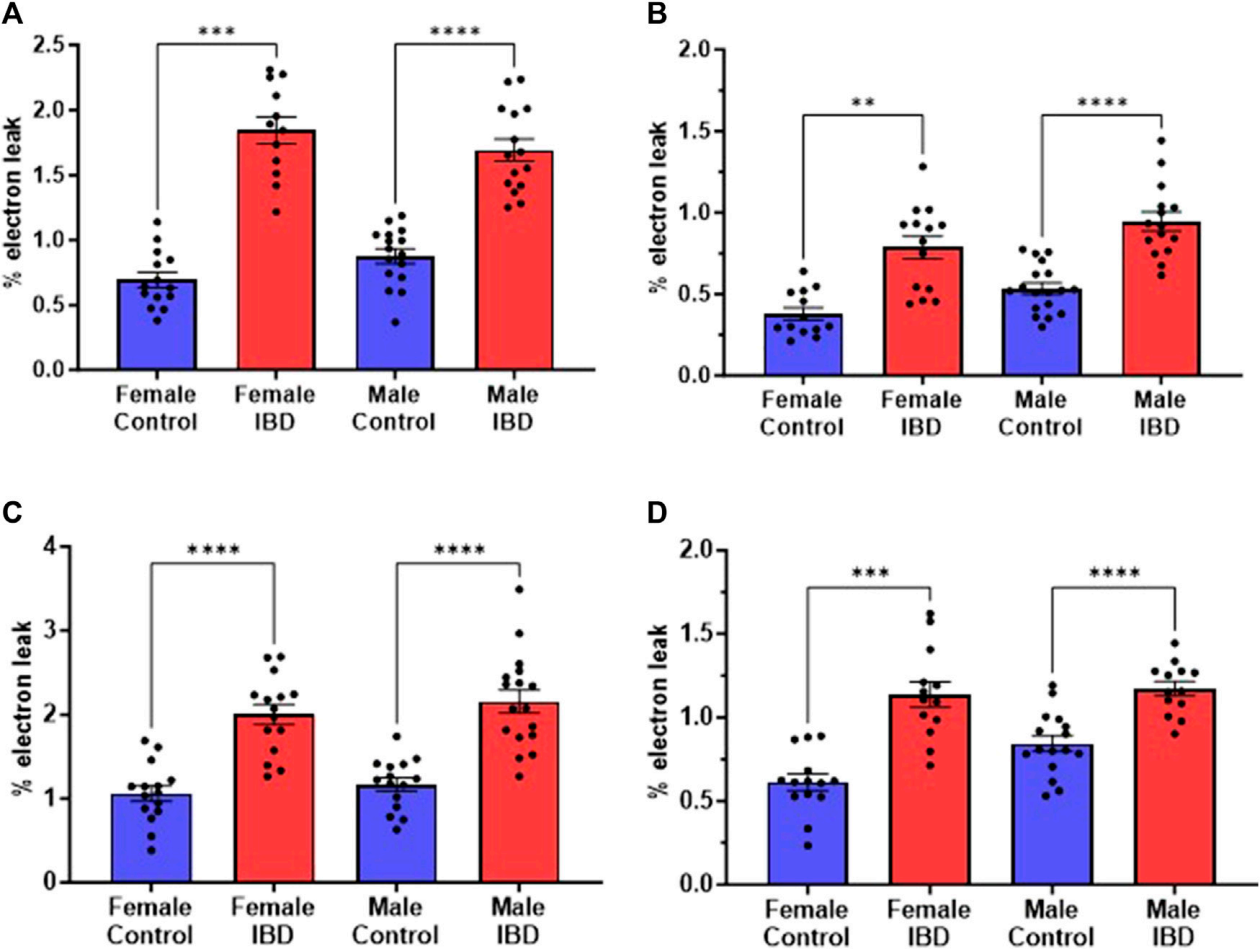
Mitochondrial reactive oxygen species (mtROS) was measured simultaneously with the respiration rate presented in Figure 3. Amplex Red was used to assess the rate of mtROS production, which is calculated as % electron leak as described in methods. During **(A)**. Complex I-driven respiration; **(B)**. Complex II-driven respiration; **(C)**. LCFA oxidation; and **(D)**. MCFA oxidation, mtROS production was significantly increased in both males and females with IBD (days 2–3) compared to controls. (** = *p* ≤ 0.01; *** = *p* ≤ 0.001; **** = *p*≤ 0.0001).

Mitochondrial ROS (mtROS) production was measured simultaneously with mitochondrial respiration. In both male and female IBD rats, mtROS was increased (Figure 4). During complex I-driven respiration (Figure 4A), complex II-driven respiration (Figure 4B), and LCFA oxidation (Figure 4C), mtROS was increased by roughly 2-fold for both IBD males and females. Using MCFA, females showed roughly a 2-fold increase in mtROS while males showed a 1.4-fold increase (Figure 4D).

### 3.5 IBD rats show alterations in individual mitochondrial enzymes

**FIGURE 5:**
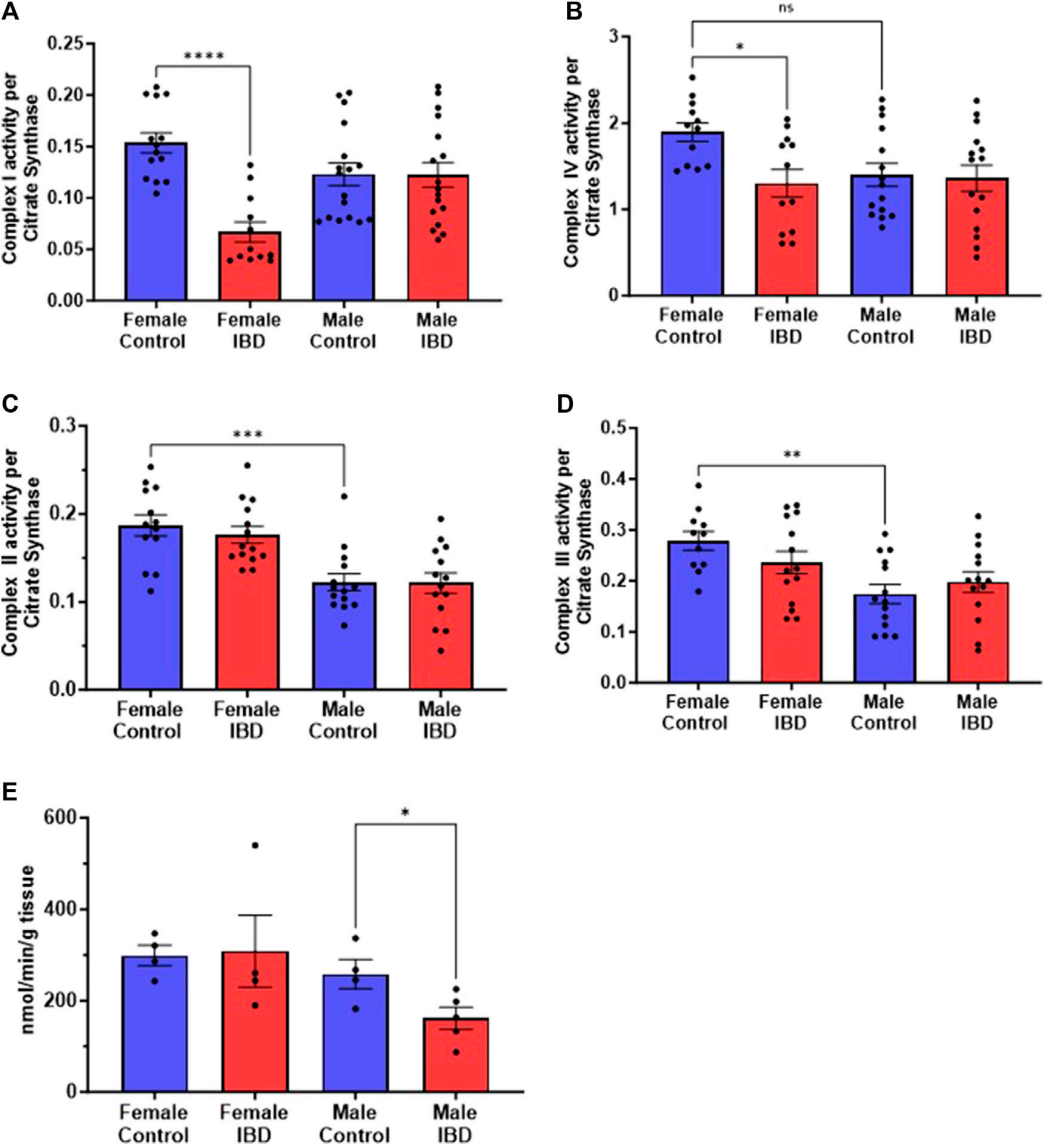
Individual mitochondrial enzyme activities were measure to determine the location of mitochondrial dysfunction. **(A)** Complex I activity was significantly decreased in IBD (days 2–3) females (*p* ≤ 0.05) compared to controls, but not in males. **(B)** Complex IV activity showed a significant decrease in IBD (days 2–3) females (*p* ≤ 0.05) compared to controls. Again, males showed a lower control activity than females (*p* ≤ 0.05). **(C)** Complex II activity showed no significant differences in IBD animals (days 2–3). However, males showed a significantly lower control activity than females (*p* ≤ 0.05). Complex activities were normalized to citrate synthase activity with final units of nmol e-/nmol citrate. **(D)** Complex III activity showed a similar trend with no change in the IBD animals (days 2–3) but with the control activity in males being significantly lower than females (*p* ≤ 0.05). **(E)** When measuring the antioxidant enzyme activities, IBD (day 2) males showed a significant decrease in catalase activity (*p* ≤ 0.05) compared to controls.

To determine what is causing the changes in colon mitochondrial function in our IBD rats, we explored the activity of the individual mitochondrial complexes of the electron transport chain and antioxidant enzymes. Colon mitochondrial complex I (Figure 5A) and complex IV (Figure 5B) activities were significantly decreased in females compared to controls with no change in complex II (Figure 5C) or III activity (Figure 5D). The IBD males showed no significant changes for any of the complexes compared to controls (Figure 5). Interestingly, female controls showed a significantly higher activity compared to males for complexes II, III, and IV (Figure 5).

To determine why the IBD males have an increase in mtROS but no change in electron transport enzymes, we explored mtROS defense mechanisms. We measured superoxide dismutase (SOD), catalase (CAT), glutathione peroxidase (GPx), glutathione S-transferase (GST), glutathione reductase (GR) and glutathione (GLT) levels, which are common components that are altered in disease states (Katerji et al., 2019). We saw no significant change in GR, GST, GPx, GLT, and SOD in both males and females (Supplementary Figure S3). However, IBD males showed a significant decrease in CAT activity (Figure 5E).

### 3.6 MitoTEMPO improves disease activity and mitochondrial function in IBD females

**FIGURE 6:**
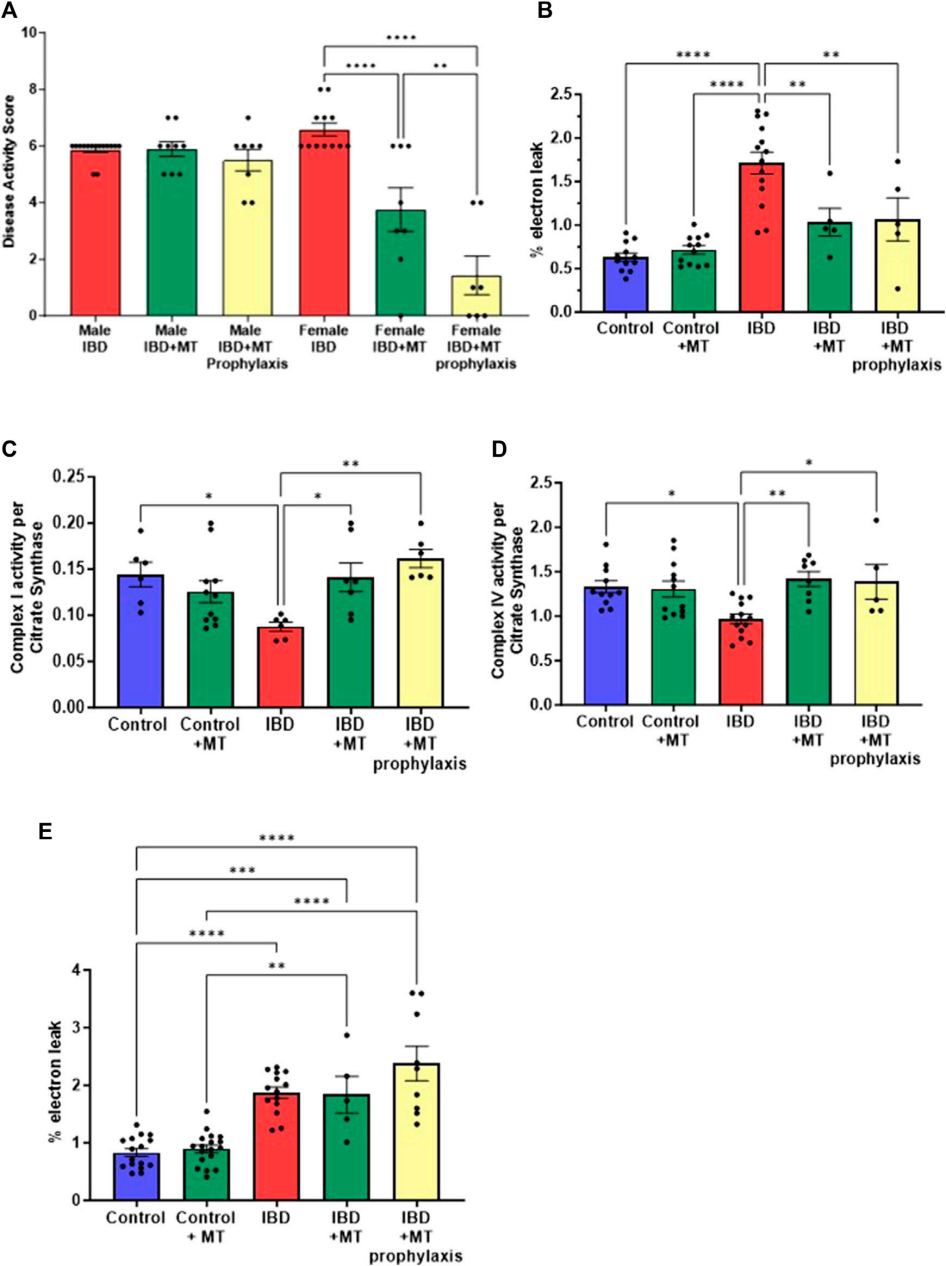
**(A)** The disease activity score was calculated as outlined in the methods. IBD animals received the mitochondrial-targeted therapy, MitoTEMPO (MT), daily following indomethacin injections until the end of the experiment (MT treatment) or daily before indomethacin injections until the end of the experiment (MT prophylaxis treatment). All data is from the peak of the disease at days 2–3. Males showed no significant change when treated with MT. However, the females saw a decrease in disease activity with MT treatment (*p* ≤ 0.05) and MT prophylaxis treatment (*p* ≤ 0.0001), with the prophylaxis treatment being more significant. **(B)** IBD females that received MT treatment (*p* ≤ 0.05) and MT prophylaxis treatment (*p* ≤ 0.01) showed a significant decrease in mtROS production (% electron leak) compared to IBD females without. **(C)** Complex I activity was increased in IBD females that received MT treatment (*p* ≤ 0.05) **(D)** Complex IV activity was increased in IBD females that received MT treatment (*p* ≤ 0.01) and MT prophylaxis treatment (*p* ≤ 0.05). Complex activities were normalized to citrate synthase activity with final units of nmol e−/nmol citrate. **(E)** MitoTEMPO treatment does not improve mtROS production (% electron leak) in IBD male rats.

With the mitochondrial dysfunction mentioned above, targeting mitochondria to improve their activity may be a potential therapeutic option. We chose to begin our exploration of mitochondrial-targeted therapies by administering mitoTEMPO as described in methods. MitoTEMPO is a mitochondrial targeted superoxide mimetic. In IBD female rats, we found that the disease activity decreased with mitoTEMPO treatment (Figure 6A). The prophylaxis protocol was the best at improving the disease. MitoTEMPO also lowered the colon mtROS production in female IBD rats (Figure 6B) and improved complex I (Figure 6C) and complex IV activity (Figure 6D). However, mitoTEMPO did not improve the disease in male rats (Figure 6A). In Figure 6E, there is no change in colon mtROS with mitoTEMPO with the IBD male rats.

### 3.7 MitoTEMPO decreases inflammatory marker levels in IBD females

**FIGURE 7:**
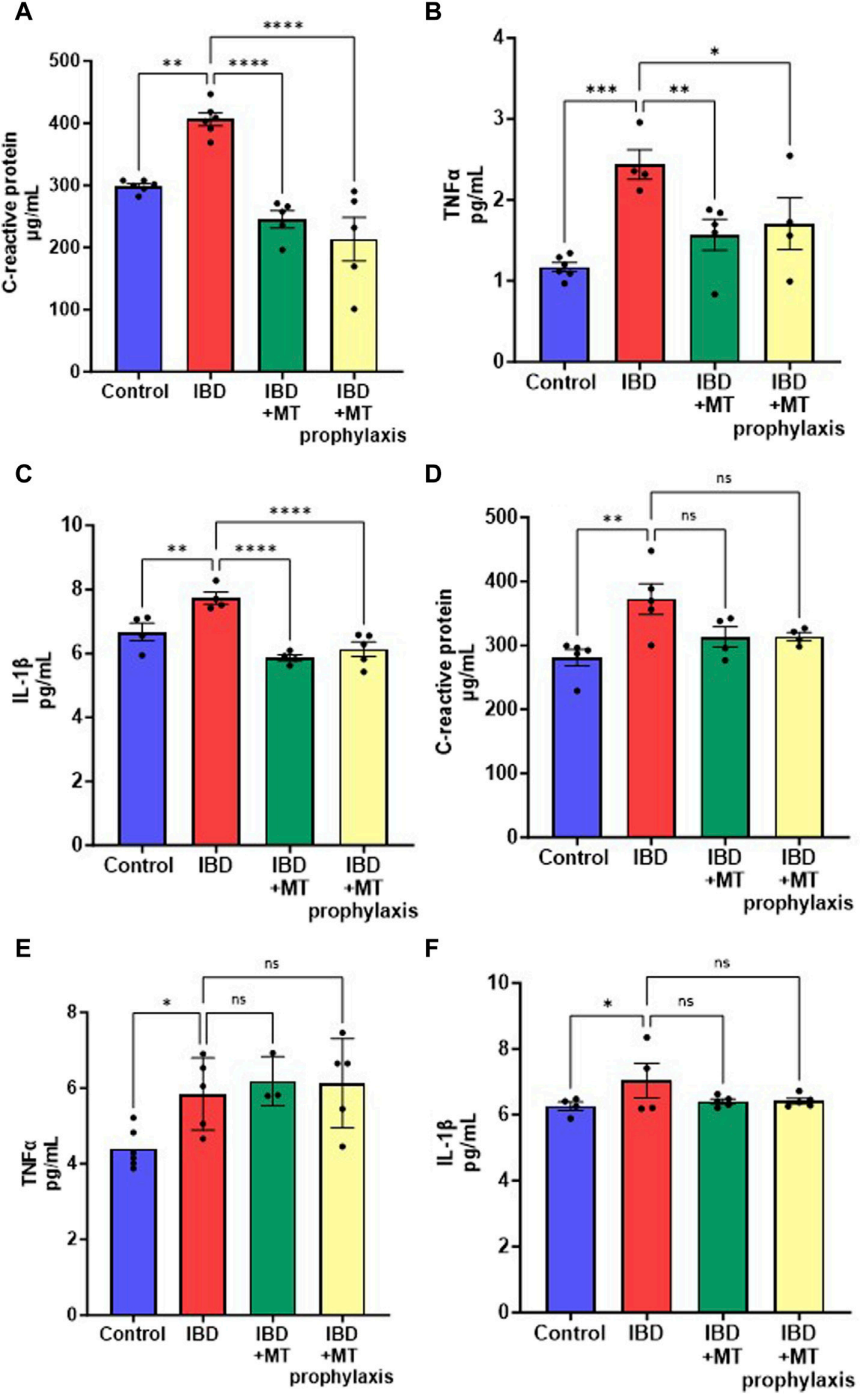
Inflammatory markers were measured to determine if mitoTEMPO could potentially improve inflammation. In IBD females **(A)** CRP, **(B)** TNFa, and **(C)** IL-1B levels significantly increased with disease but are significantly decreased with both mitoTEMPO treatment protocols. In the IBD males, **(D)** CRP, **(E)** TNFa, and **(F)** IL-1B levels significantly increased with disease but no significant change with mitoTEMPO treatments. (* = *p* ≤ 0.05; ** = *p* ≤ 0.01; *** = *p* ≤ 0.001; **** = *p* ≤ 0.0001).

To further determine if mitoTEMPO has the potential to improve inflammation. We measured serum levels of c-reactive protein (CRP) and the inflammatory cytokines TNFα and IL-1β. In the female rats with IBD, we saw a significant decrease in CRP (Figure 7A), TNFα (Figure 7B), and IL-1β (Figure 7C) with both the treatment and prophylaxis protocol. As with the disease activity score, we saw no significant change in inflammatory markers with the IBD male rats that received both mitoTEMPO treatments (Figures 7D–F).

## 4 Discussion

Clinical studies have shown sex differences in onset, severity, and extra intestinal manifestation in IBD (Xu et al., 2018; MKoma, 2013; Goodman et al., 2020). This is likely due to the hormonal change and studies have shown at puberty there is an increase in IBD diagnosis in females than males. We chose to use rats at the age of puberty to explore this effect in the indomethacin induced IBD rat model. We found that two injections 24 h apart induced acute GI inflammation resulting in weight loss 2 days after injections in both male and female rats. IBD female rats showed a more significant loss in body weight, a significant increase in colon hypertrophy, and slightly higher disease activity score compared to IBD male rats. These results indicate that there are slight differences in the severity of the disease between males and females, which is similar to the observed clinical variations between males and females.

Mitochondrial function is important to maintaining epithelial barrier function (Berger et al., 2016). To begin our exploration into mitochondrial function in our IBD model, we measured mitochondrial content as either citrate synthase activity or cardiolipin levels. Cardiolipin has been reported as the most reliable measure of mitochondrial content followed by citrate synthase (Larsen et al., 2012). What we have demonstrated here is that both measures show the same trend. Due to these similarities, we used citrate synthase activity to normalize to mitochondrial content. IBD male rats have a significant decrease in colon mitochondrial content compared to controls while females showed no change in mitochondrial content in the IBD rats. These results could suggest that the males have improved mitophagy mechanisms compared to females, which will be tested in future studies.

Mitochondrial oxidative phosphorylation (OXPHOS) is important for generating ATP. ATP production is essential to maintaining epithelial barrier function. Electrons enter the electron transport chain through two different pathways. One pathway is by the oxidation of NADH by complex I. The other is the oxidation of succinate to fumarate by complex II. Both complex I and II will transfer electrons to a membrane bound electron acceptor ubiquinone to generate ubiquionol. Electrons are then transferred through complex III to cytochrome *c*. Cytochrome *c* will bind and transfer an electron to complex IV, which ultimately transfers electrons to oxygen to generate water. Complex I, III, and IV are proton pumps which pump protons from the matrix to the inner membrane space to generate a proton motive force that is utilized by ATP synthase to convert ADP to ATP. Defects in OXPHOS have been implicated in various diseases, especially those related to inflammation (Missiroli et al., 2020). Respiratory control ratios (RCRs) are an indication of how efficiently the mitochondria are generating ATP. Female IBD rats showed a significant decrease in colonic mitochondrial RCR values. When normalized to citrate synthase, only the female IBD rats showed a decrease in intact colonic mitochondrial respiration. These results indicate that protons are leaking through the inner mitochondrial membrane by a pathway other than ATP synthase, resulting in lower ATP production and lower OXPHOS capacity. The loss of ATP in IBD female colon mitochondria will impair the ability of epithelial cells to maintain barrier function resulting in GI inflammation (Ballard and Towarnicki, 2020).

When mitochondrial oxidative phosphorylation is decreased, mitochondrial ROS (mtROS) production is increased. This occurs through the production of superoxide, which will contribute to cellular oxidative stress and is linked to diseases and epithelial barrier dysfunction (Kimmich, 1970; Ballard and Towarnicki, 2020). Both male and female IBD rats showed a significant increase in the production of mtROS compared to control regardless of substrate. This increase in mtROS will contribute to the gut dysbiosis, lower ATP production by the epithelial cells, and epithelial barrier dysfunction resulting in GI inflammation (Ballard and Towarnicki, 2020). There are two significant locations for mtROS production from OXPHOS, complex I and complex III. Complex I will produce mtROS when the FMN site is reduced to FMNH_2_ leading to a high NADH/NAD^+^ ration, which increases the likelihood that electrons will be transferred to O_2_ instead of through the iron-sulfur centers (Murphy, 2009). Complex I and III will produce mtROS if there is an increase in the highly reactive semiquionone, which will quickly react with O_2_ to produce mtROS (Murphy, 2009). Any inhibition in the electron transport chain will result in an increase in the reduction of these intermediates that will increase the probability of mtROS production. In the IBD female colon mitochondria, we found that complex I and IV activities are decreased, which would lead to an increase in both FMH_2_ and the semiquinone, ultimately leading to the increase in mtROS.

Although mtROS may be produced there is a balance between antioxidants and mtROS. However, when the balance between antioxidants and ROS species is disrupted, oxidative stress can occur (Katerji et al., 2019). Assessment of the antioxidant status is correlated to the extent of oxidative stress. Redox homeostasis is regulated either enzymatically or non-enzymatically. Superoxide dismutase, catalase, glutathione peroxidase, glutathione S-transferase, glutathione reductase and glutathione levels are common components that are altered in disease states (Katerji et al., 2019). When we explored these antioxidant mechanisms, the only one that showed a significant difference was catalase. Catalase catalyzes the decomposition of hydrogen peroxide to water and oxygen (Sharma and Ahmad, 2014). In colons from male IBD rats, we found that catalase was significantly decreased. The amplex red assay measures the presence of H_2_O_2_. Therefore, since the male IBD rats have a decrease in catalase activity there will be an increase in H_2_O_2_.

Based on these results, using a mitochondrial-targeted therapy is a feasible option for treatment. MitoTEMPO is a mitochondria-targeted superoxide dismutase mimetic that possesses superoxide and alkyl radical scavenging properties (Dikalov, 2011). This compound combines the antioxidant piperidine nitroxide TEMPO with the lipophilic cation triphenylphosphonium, which allows it to pass through lipid bilayers and accumulate in mitochondria (Dikalov, 2011). Mitochondrial targeting of superoxide scavenging via mitoTEMPO has been examined for potential therapeutic benefit to a variety of mitochondrial dysfunctions arising from excessive reactive oxygen species (Liang et al., 2009; Dikalova et al., 2010; Liang et al., 2010). IBD disease activity and inflammation was improved with mitoTEMPO treatment in female rats but not males. As expected mitoTEMPO decreased mtROS in the colon of IBD females rats and it also improved the activity of complex I and IV. However, mitoTEMPO increased mtROS in males. This is likely due to mitoTEMPO converting superoxide to H_2_O_2_. However, the males have lower catalase activity leading to an increase in the levels of H_2_O_2_. A mitochondrial-targeted catalase mimetic will potentially be a better treatment option for the males but creating a catalase mimetic can be challenging. However, a study in 2016 designed a nanosystem based on Tempol and a biocompatible B-cyclodextrin-derived material (Zhang et al., 2016). The Tempol serves as a SOD mimetic, while the nanocontainer is mainly composed of a hydrogen peroxide-eliminating material that functions as a catalase-mimicker (Zhang et al., 2016). A treatment like this may work better for our male IBD colon mitochondrial function and will be explored in the future. Another feasible option is the supplementation with MCFAs. MCFAs have been shown to lower mtROS in other disease models (Edwards et al., 2018). In this study, the males produce less mtROS with MCFA than other mitochondrial substrates. We will also explore other mitochondrial-targeted therapies in the future.

While both mitoTEMPO treatment methods are significantly different from the IBD females, the treatment methods are also significantly different. However, when looking at the mtROS production and the complex I and IV activities there is no difference between the treatment methods. MitoTEMPO is a mitochondria-targeted superoxide dismutase mimetic that will decrease the concentration of superoxide locally in the mitochondria. This could explain the lack of difference between the mtROS production and the complex I and IV activities. Between the first and second indomethacin injection it is possible that mtROS production increases and escapes the mitochondria to contribute to the disease induction and inflammation but damage to the enzymes has not occurred at this point. In the future, we would like to explore when the onset of mitochondrial dysfunction occurs during the induction of the disease, the correlation to disease activity, and the timing of treatment with mitoTEMPO. Additionally, we plan to explore the relationship between mitochondrial dysfunction, gut dysbiosis, and GI inflammation.

Interestingly, our findings also highlight sex differences in colon mitochondrial function. There have been several studies exploring the effect of sex hormones on mitochondrial function (Sultanova et al., 2020). Estrogen acts through the estrogen receptor β (ERβ) to increase mitochondrial DNA (mtDNA)–encoded transcription of cytochrome oxidase subunits I, III, and IV; increase respiration capacity; elevate antioxidant activity; and inhibit apoptosis (Liao et al., 2015). Studies have shown that an increase in the androgen receptor (AR) negatively regulates the expression, assembly, integrity, and function of individual mitochondrial complexes resulting in decreased mitochondrial OXPHOS (Bajpai et al., 2019). The animals used in this study are at the age of puberty for rats. Therefore, sex hormones are on the rise, which will increase the chances of observing sex differences. When comparing control males and females, we observed differences in colon mitochondrial function. Control males showed a significant decrease in complex I-driven respiration, complex II-driven respiration and MCFA oxidation compared to control females. Control males also showed a significant decrease in complex II, III, and IV activity compared to control females. This highlights the importance of identifying sex differences in mitochondrial function in disease models. In future studies, we will alter the sex hormone status in our IBD animals to further determine the effects of sex hormones on mitochondrial function.

In conclusion, we have shown that female SD rats have an increase in disease severity compared to males. We have also demonstrated that there are differences in colon mitochondrial function between male and female SD rats. This may contribute to the differences in disease severity. While both male and female rats have increases in mtROS production, the cause for the increase is different between males and females. In females, complex I and IV have decreased activity resulting in the potential for increased mtROS. In males, catalase activity is decrease which will result in an increase of H_2_O_2_. Targeting mitochondria with the superoxide mimetic (mitoTEMPO) improves the disease activity in females, decreases mtROS, and improves complex I and IV activity. However, it did not prove to be a viable treatment option for males.

## Data Availability

The original contributions presented in the study are included in the article/Supplementary Material, further inquiries can be directed to the corresponding author.

## Abbreviations

CAT, catalase; CD, Crohn’s disease; CRC, colorectal cancer, DSS, dextran sulfate sodium; GI, gastrointestinal; GPx, glutathione peroxidase; GR, glutathione reductase; GST, glutathione s-transferase; IBD, inflammatory bowel disease; LCFA, long-chain fatty acid; MCFA, medium-chain fatty acid; mtROS, mitochondrial reactive oxygen species; OXPHOS, oxidative phosphorylation, PAMPs, pathogen-associated molecular patterns; RCR, respiratory control ratio; SO, superoxide; SOD, superoxide dismutase; UC, ulcerative colitis.
