# Supplementary figures and images for: Sex-specific colonic mitochondrial dysfunction in the indomethacin-induced rat model of inflammatory bowel disease
Source: Front Physiol. 2024 Mar 26;15:1341742. doi: 10.3389/fphys.2024.1341742 (PMC11002206; doi:10.3389/fphys.2024.1341742)

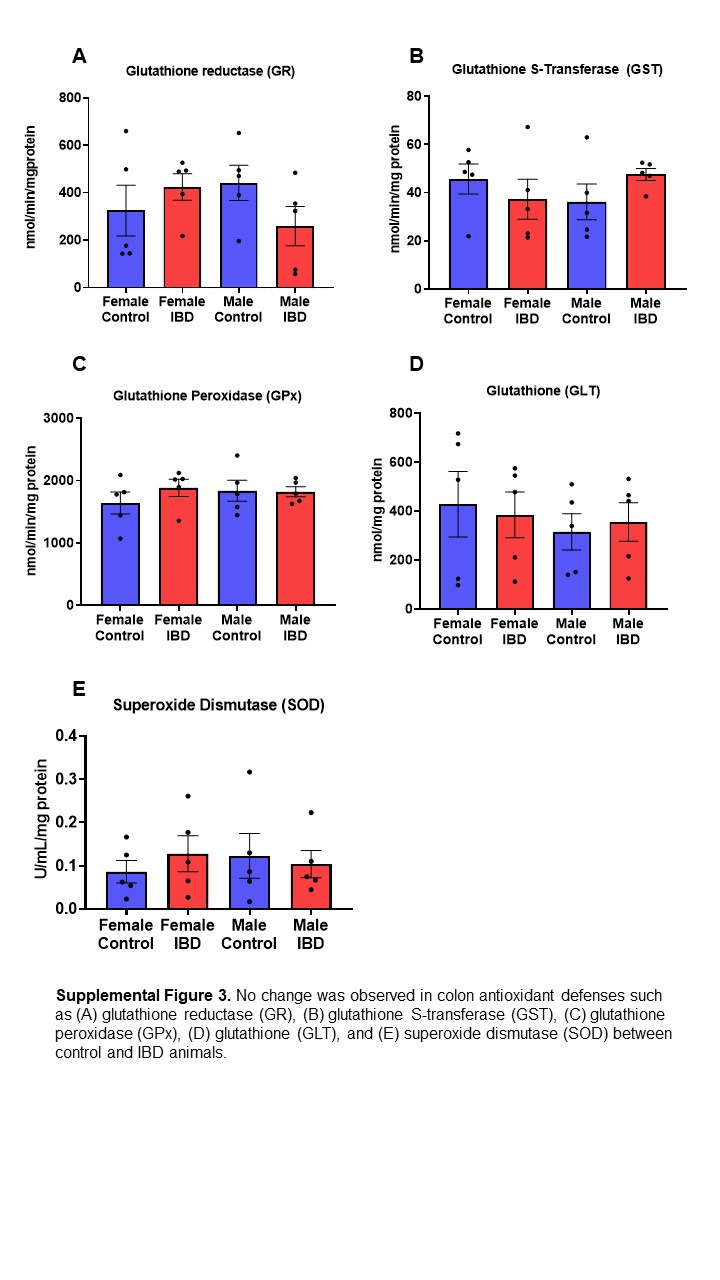

Supplement: Supplementary file 1 [file Image3.JPEG]

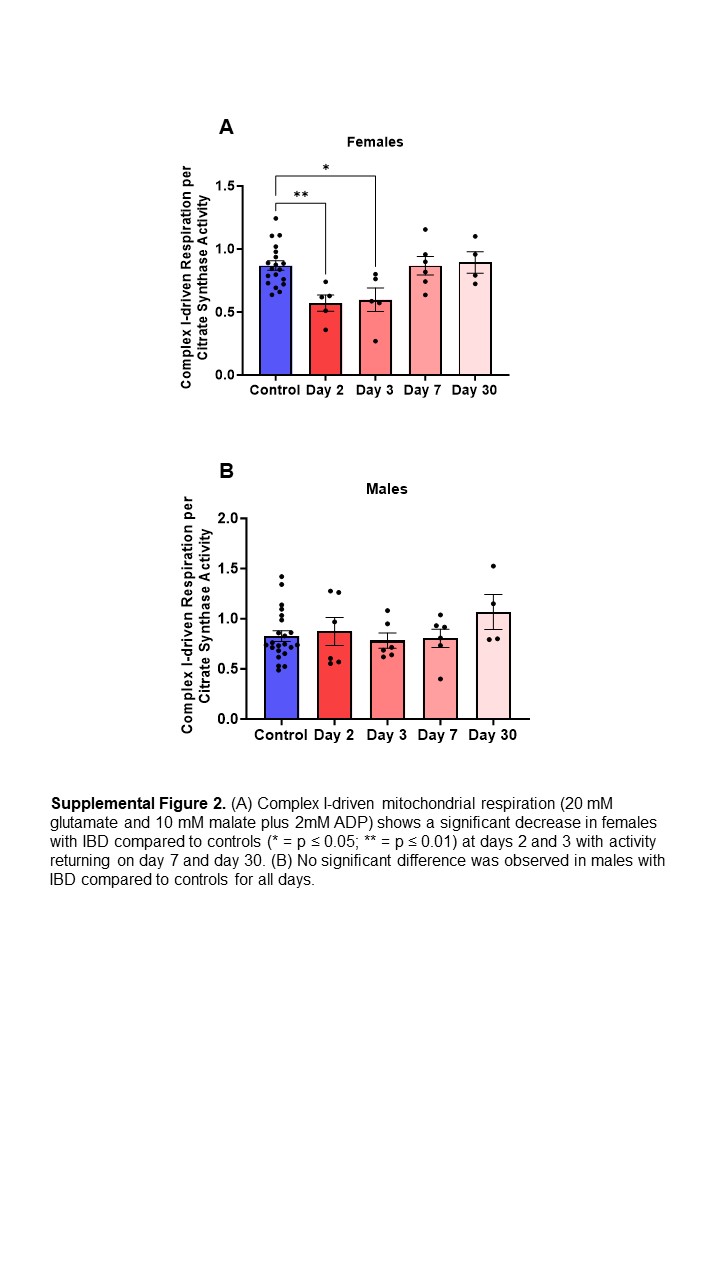

Supplement: Supplementary file 2 [file Image2.JPEG]

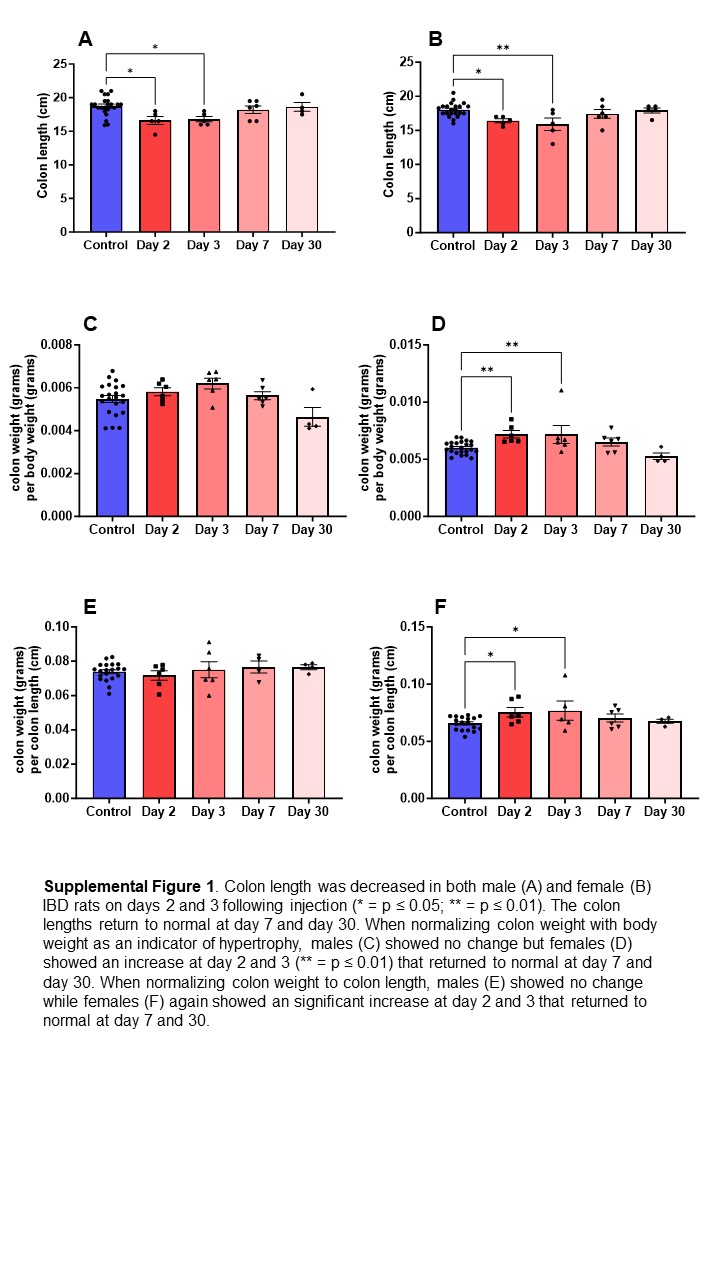

Supplement: Supplementary file 3 [file Image1.jpg]
